# Integrating Machine Learning and Molecular Methods for *Trichophyton indotineae* Identification and Resistance Profiling Using MALDI-TOF Spectra

**DOI:** 10.3390/pathogens14100986

**Published:** 2025-09-30

**Authors:** Vittorio Ivagnes, Elena De Carolis, Carlotta Magrì, Manuel J. Arroyo, Giacomina Pavan, Anna Cristina Maria Prigitano, Anuradha Chowdhary, Maurizio Sanguinetti

**Affiliations:** 1 Dipartimento di Scienze di Laboratorio ed Ematologiche, Fondazione Policlinico Universitario “A. Gemelli” IRCCS, 00168 Rome, Italy; vittorio.ivagnes@gmail.com (V.I.); carlottamagri97@gmail.com (C.M.); maurizio.sanguinetti@unicatt.it (M.S.); 2 Clover Bioanalytical Software S.L., 18016 Granada, Spain; manuel.arroyo@cloverbiosoft.com; 3 Department of Microbiology, San Bortolo Hospital, 36100 Vicenza, Italy; giacomina.pavan@aulss8.veneto.it; 4 Department Biomedical Sciences for Health, Università degli Studi di Milano, 20122 Milano, Italy; anna.prigitano@unimi.it; 5 Medical Mycology Unit, Department of Microbiology, Vallabhbhai Patel Chest Institute, University of Delhi, Delhi 110007, India; chowdhary.anuradha@gmail.com

**Keywords:** *Trichophyton indotineae*, dermatophytosis, terbinafine resistance, squalene epoxidase, MALDI-TOF MS, machine learning, fungal diagnostics, antifungal susceptibility testing, biomarker discovery

## Abstract

*Trichophyton indotineae* is an emerging dermatophyte species responsible for recalcitrant and terbinafine-resistant dermatophytosis, raising concerns over diagnostic accuracy and treatment efficacy. This study aimed to improve the identification and resistance profiling of *T. indotineae* by integrating molecular methods with machine learning-assisted analysis of MALDI-TOF mass spectra. A total of 56 clinical isolates within the *Trichophyton mentagrophytes* complex were analyzed using ITS and *ERG1* gene sequencing, antifungal susceptibility testing, and MALDI-TOF MS profiling. Terbinafine resistance was detected in 23 isolates and correlated with specific *ERG1* mutations, including F397L, L393S, F415C, and A448T. While conventional MALDI-TOF MS failed to reliably distinguish *T. indotineae* from closely related species, unsupervised statistical methods (PCA and hierarchical clustering) revealed distinct spectral groupings. Supervised machine learning algorithms, particularly PLS-DA and SVM, achieved 100% balanced accuracy in species classification using 10-fold cross-validation. Biomarker analysis identified discriminatory spectral peaks for both *T. indotineae* and *T. mentagrophytes* (3417.29 *m*/*z* and 3423.53 *m*/*z*). These results demonstrate that combining MALDI-TOF MS with multivariate analysis and machine learning improves diagnostic resolution and may offer a practical alternative to sequencing in resource-limited settings. This approach could enhance the routine detection of terbinafine-resistant *T. indotineae* and support more targeted antifungal therapy.

## 1. Introduction

Dermatophytosis is one of the most prevalent fungal infections worldwide, affecting millions of individuals annually [1]. It is caused by dermatophytes, a group of keratinophilic fungi that invade keratinized tissues such as skin, hair, and nails. The increasing global burden of dermatophytosis, particularly in regions with high humidity and dense populations, has raised significant concerns regarding treatment efficacy, antifungal resistance, and the emergence of new pathogenic species [2,3]. Among the dermatophytes, *T. mentagrophytes* and *Trichophyton rubrum* have historically been considered as the most common causative agents of dermatophytosis [4,5]. However, in recent years, a new species, *T. indotineae*, has been recognized as a distinct dermatophyte within the *T. mentagrophytes* complex and is now a major etiological agent of recalcitrant and drug-resistant infections [6,7]. Recent phylogenetic studies have highlighted the complexity of the *T. mentagrophytes*/*T. interdigitale* species boundaries [8,9], and multilocus as well as genomic approaches have been proposed to better resolve the taxonomy of dermatophytes [10,11].

The emergence of *T. indotineae* is particularly concerning due to its increasing prevalence and its ability to cause chronic, severe, and widespread skin infections, which are often refractory to conventional antifungal treatments [12,13]. Initially reported in South Asia, particularly in India, where cases of terbinafine-resistant dermatophytosis have surged, *T. indotineae* has since been detected in Europe, the Middle East, and other regions, suggesting a potential for global spread [7]. Epidemiological investigations confirm that *T. mentagrophytes*/*T. interdigitale* infections remain highly prevalent in Europe [14], with evidence of animal reservoirs contributing to the spread of genotype V [15]. In India, large-scale analyses have shown major shifts in the dermatophyte spectrum over recent decades [16]. More recently, *T. indotineae* has been reported in Europe, including Hungary [17], supporting previous observations of changing epidemiological trends worldwide [18]. The widespread and often inappropriate use of topical antifungals and corticosteroids has contributed to the selective pressure favoring resistant strains, resulting in persistent and difficult-to-treat infections. Unlike other dermatophytes, *T. indotineae* is frequently associated with deep and extensive tinea corporis and tinea cruris infections, often presenting as erythematous, scaly, and hyperkeratotic lesions with inflammation and significant discomfort for patients [19].

One of the key differences between *T. indotineae* and *T. mentagrophytes* lies in their genetic composition and antifungal susceptibility profiles. While *T. mentagrophytes* has long been considered a major cause of dermatophytosis, it remains largely susceptible to terbinafine, an allylamine antifungal that inhibits squalene epoxidase, a key enzyme in ergosterol biosynthesis coded by the *ERG1* gene [20,21]. In contrast, *T. indotineae* exhibits high rates of terbinafine resistance, making it a major therapeutic challenge [6]. The primary mechanism of resistance in *T. indotineae* is attributed to point mutations in the *ERG1* gene, which reduce the drug’s binding affinity and impair its fungicidal action. This enzyme, originally characterized in *Saccharomyces cerevisiae* through terbinafine-sensitive variants [22], plays a central role in ergosterol biosynthesis, and amino acid substitutions reduce terbinafine’s binding affinity. Several *ERG1* mutations have been identified, with the most common being F397L, L393S, F415C, and A448T, which confer varying levels of resistance [23,24,25,26]. Strains carrying the F397L and L393S mutations exhibit high MIC values (often ≥ 32 µg/mL), making terbinafine ineffective.

The identification of *T. indotineae* remains a significant challenge, particularly in clinical laboratories where conventional diagnostic methods may fail to differentiate it from closely related species [12,27]. ITS (internal transcribed spacer) sequencing has been established as the gold standard for molecular identification, but it is not routinely available in many diagnostic settings. MALDI-TOF MS (matrix-assisted laser desorption ionization–time of flight mass spectrometry), a widely used technique for fungal identification, has shown limited reliability in distinguishing *T. indotineae* from *T. mentagrophytes* [11,28]. The high genetic similarity between the two species leads to spectral overlap, resulting in misidentification or ambiguous results [29]. The limitations of current MALDI-TOF MS libraries in differentiating *T. indotineae* from closely related species may be overcome by updated databases such as the MSI-2 application, which has been shown to reliably identify *T. indotineae* [28]. This underscores the critical need to implement updated MALDI-TOF MS databases reflecting the latest taxonomic revisions, ensuring accurate identification of closely related species such as *T. indotineae* and *T. mentagrophytes* [30]. Without integrating the new taxonomy into diagnostic platforms, even advanced identification tools risk perpetuating diagnostic errors and hindering appropriate clinical management. This has led to an increased reliance on molecular methods, such as *ERG1* sequencing and phylogenetic analysis, to accurately differentiate *T. indotineae* and assess its resistance profile. In this context, an SYBR Green-based real-time PCR assay has recently been developed and validated for the rapid and specific identification of *T. indotineae*, offering an additional molecular tool for clinical laboratories [31]. However, these methods require specialized equipment and expertise in melting curve analysis, besides additional costs due to DNA extraction, limiting their widespread implementation.

In this study, we conducted a comprehensive analysis of *T. indotineae* and *T. mentagrophytes* isolates, including terbinafine susceptibility testing, molecular identification via ITS and *ERG1* sequencing, and MALDI-TOF MS spectral analysis. Additionally, we employed PCA (Principal Component Analysis) and hierarchical clustering to assess whether advanced statistical modeling could enhance the discriminatory power of MALDI-TOF MS in distinguishing *T. indotineae* from related species and if, thanks to integration between machine learning and MALDI-TOF mass spectra profiles, this might be helpful for fast and accurate detection at the species level of this emerging resistant fungus.

## 2. Materials and Methods

### 2.1. Sample Collection

A total of 56 clinical samples of *T. mentagrophytes* (*n* = 33) and *indotineae* (*n* = 23) harvested from skin scales were collected from various origins, including outpatients at Fondazione Policlinico Universitario “A. Gemelli” IRCCS in Rome, San Bortolo Hospital in Vicenza, Italy, and Vallabhbhai Patel Chest Institute, Delhi (Table S1). All patients reported redness, itching, and typical ring-like shaped skin patches from which material was collected by scraping. Fluorescence microscopy of KOH-digested clinical specimens was routinely performed in all the centers. All samples were cultivated onto Sabouraud dextrose agar (SDA) (Vacutest Kima S.r.l., Arzergrande, Italy) at 30 °C for up to two weeks [32,33]. Upon mycelial growth revealing typical *Trichophyton* spp.’s white colony appearance along with cream to yellowish reverse, lactophenol–cotton blue staining was executed. Branched, septate hyphae with spiral forms or rare club-shaped macroconidia were observed; subsequently, MALDI-TOF analysis, DNA extraction, and sequencing of the rDNA internal transcribed spacer (ITS) was performed for identification at the species level.

### 2.2. Terbinafine Susceptibility

Following the EUCAST reference method for antifungal susceptibility testing of microconidia-forming dermatophytes (E.Def 11.0) [34], isolates were subcultured on Sabouraud dextrose agar (SDA) supplemented with cycloheximide (300 mg/L) and chloramphenicol (50 mg/L) and incubated at 25–28 °C for 4–7 days to obtain sufficient microconidia. A stock powder of terbinafine was dissolved in dimethyl sulfoxide (DMSO), and serial twofold dilutions were prepared in RPMI 1640 broth supplemented with 2%, covering a concentration range of 0.125–32 mg/L.

To prepare the inoculum, colonies were covered with approximately 5 mL of sterile distilled water containing 0.1% Tween 20. The microconidia were gently dislodged using a sterile cotton swab, transferred to a sterile tube, vortexed for 15 s at ~2000 rpm, and filtered through a sterile 11 µm pore-size filter to remove hyphal fragments. The suspension was adjusted to 2 × 10^6^–5 × 10^6^ conidia/mL by counting the microconidia in a hemocytometer chamber and then diluted 1:10 with sterile distilled water to yield a working inoculum of 2 × 10^5^–5 × 10^5^ CFU/mL. Flat-bottom tissue culture-treated 96-well plates were prepared by dispensing 100 µL of antifungal drug dilution per well along with an equal volume (100 µL) of inoculum suspension, resulting in a final inoculum concentration of 1 × 10^5^–2.5 × 10^5^ CFU/mL. Growth control wells (without drug) and sterility control wells (medium only) were included in each plate.

Plates were incubated at 25–28 °C without agitation and read after 5 days. MICs were determined visually as the lowest drug concentration producing a ≥50% reduction compared to the growth control. According to EUCAST tentative epidemiological cut-off values (ECOFFs), terbinafine MICs of ≤0.125 mg/L classify isolates of *T. indotineae* as wild type [34,35].

### 2.3. Sequencing of the ITS Region and the ERG1 Gene

Molecular identification of fungal isolates was performed by sequencing the partial ITS region, a highly conserved genomic marker for fungal species differentiation. DNA was extracted using the DNeasy Plant Mini Kit (Qiagen, Hilden, Germany), and PCR was performed with the primer pair 5′-TCCGTAGGTGAACCTGCGG-3′ (forward) and 5′-TCCTCCGCTTATTGATATGC-3′ (reverse) [31]. The thermal cycling conditions consisted of an initial denaturation at 95 °C for 15 min, followed by 32 cycles of denaturation at 95 °C for 30 s, annealing at 55 °C for 30 s, and extension at 72 °C for 45 s, with a final elongation step at 72 °C for 3 min and cooling at 4 °C. The resulting PCR products were purified using the MinElute PCR Purification Kit (Qiagen, Hilden, Germany) and analyzed with Chromas software (Version 2.6.6). Identification was confirmed by NCBI BLAST (https://blast.ncbi.nlm.nih.gov/Blast.cgi), and results were deemed reliable if the sequence identity exceeded 98%. Key reference sequences included *T. mentagrophytes* (MF926358) and *T. indotineae* (OR417031.1). Multiple sequence alignment of the ITS and *ERG1* sequences was made by ClustalW using MEGA11 software (Version 11.0.10) [36,37,38,39].

For ITS typing, a phylogenetic tree was constructed using the neighbor-joining (NJ) method, and the evolutionary distances were computed using the Tamura–Nei parameter. The sequences were aligned with the reference FASTA ITS sequences downloaded at https://github.com/Ivan-Pchelin/genotyping-by-sequencing/blob/master/referenceset.fasta (accessed on 11 September 2025). The amplification of the *ERG1* gene was carried out using the KAPA HiFi HotStart ReadyMix PCR Kit (Roche, Basel, Switzerland), which contains a high-fidelity engineered B-family DNA polymerase for superior accuracy and sensitivity. PCR was performed on the same DNA extracted for the sequencing ITS region with the primer pair 5′-AGCTGGCAGACTTCCTTTATC-3′ (forward) and 5′-GCAGAGATAATGCAGCCACC-3′ (reverse). The thermal cycling conditions consisted of an initial denaturation at 95 °C for 3 min, followed by 25 cycles of denaturation at 98 °C for 20 s, annealing at 64 °C for 15 s, and extension at 72 °C for 90 s, with a final elongation step at 72 °C for 2 min and cooling at 4 °C. Amplified products were visualized on a 2% agarose gel stained with Midori Green Advance (Nippon Genetics, Tokyo, Japan) to confirm successful amplification. The amplified PCR products were purified using the MinElute PCR Purification Kit (Qiagen, Hilden, Germany) and sequenced using the primers 5′-GTCACCATTGTCGAGACCAAG-3′, 5′-TATGCCTCTACGTTCCGAAAG-3′, 5′-GATTGATGTTCCTAGGTGACT-3′, and 5 ′-CGGTATGACCGTGGCATTTAA-3′ to detect mutations associated with antifungal resistance, including F397L and L393F [40]. The obtained sequences were aligned and compared with reference sequences in GenBank using MEGA11 software v. 11.0.10 alignment tools.

*T. indotinae* ITS and *ERG1* gene sequences were deposited in GenBank under the accession numbers PX359035–PX359057, PX363674–PX363677, and PX392169–PX392191.

### 2.4. MALDI-TOF Spectra

Mass spectra were acquired using an Autof MS2600 (Autobio Diagnostics, Zhengzhou, China) system to evaluate its capability in distinguishing *Trichophyton* species. The spectra were obtained following standard procedures for fungal protein extraction [41] and analyzed with Autof acquirer software package V2.0.196 and the profiles matched against the Autobio library V1120. The same spectral raw data were further processed and analyzed using Clover Mass Spectrometry Data Analysis Software (MSDAS) v1.10.0 (Clover Bioanalytical Software S.L., Granada, Spain), a machine learning and multivariate analysis online platform. Preprocessing steps included variance stabilization, baseline subtraction using the Tophat filter (0.02), and smoothing with the Savitzky–Golay filter (window length: 11; polynomial order: 3). Replicates were grouped to create an average spectrum per isolate, followed by alignment (constant tolerance: 2 Da; linear tolerance: 600 ppm). The processed spectra were binned at 0.5 Da intervals and were then subjected to PCA and hierarchical clustering to assess spectral differences and determine whether clustering patterns could distinguish between the two species. PCA was performed after scaling the data to identify variance in the dataset, while hierarchical clustering (HC) grouped the spectra based on spectral similarities. A prior PCA using 28 components, which accounted for 95.63% of the total variance, was applied before performing HC. Euclidean and Ward’s methods were used as the distance metric and linkage method, respectively. The optimal cut-off was calculated based on Simpson’s Diversity index (SDI) and mean coherence (mC) for the species.

For the supervised algorithms, the same processed binned spectra normalized using the Total Ion Count (TIC) method were used as input data to train Random Forest (RF), LightGBM, Support Vector Machine (SVM), Partial Least Squares Discriminant Analysis (PLS-DA), and K-Nearest Neighbors (KNN) models to discriminate *T. mentagrophytes* from *T. indotineae* species. Hyperparameters (Table S2) were optimized via a cross-validated grid search over a parameter grid for all algorithms to optimize balanced accuracy, using *T. indotineae* as the positive category with a score threshold of 0.5 except for PLS-DA, for which three components were chosen. A K-fold cross-validation method (k = 10) was used as internal validation, as described previously [42,43], to calculate the balanced accuracy. A biomarker analysis was also performed in the Clover MSDAS platform, in which potential biomarkers were searched for both species by analyzing the peak Receiver Operating Characteristic (ROC) curves and *t*-test as univariate analysis, in which a-values (FDR-adjusted *p*-values) were calculated for each peak. Peaks analyzed were selected by applying an intensity threshold of 0.01 and a signal–noise ratio (SNR) of 2 dB. Tolerances for peaks were set to 1 Da as a constant mass tolerance and 300 ppm as a linear mass tolerance.

## 3. Results

### 3.1. Isolate Molecular Identification

By complete ITS sequencing, isolates were identified as *T. mentagrophytes* (n = 33) and *T. indotineae* (n = 23). In particular 29 *T. mentagrophytes* isolates showed 100% identity to publicly available DNA sequence acc. n. MF926358 (genotype III*). The remaining ITS sequences of the isolates 538 and TMV-FPG (genotype XXVI), 1267-FPG, and 1106-FPG were deposited in GenBank under the accession numbers PX363674–PX363677 and included in the phylogenetic tree (Figure S1).

All *T. indotineae* isolate partial ITS sequences were deposited in GeneBank under the accession numbers PX359035–PX359057.

### 3.2. Terbinafine Susceptibility

A total of 56 clinical isolates, comprising *T. mentagrophytes* (n = 33) and *T. indotineae* (n = 23), were subjected to terbinafine susceptibility testing, with molecular identification confirmed by sequencing. All 33 *T. mentagrophytes* isolates were susceptible to terbinafine. Among the *T. indotineae* isolates, six showed MIC values above the wild-type limit (>32 mg/L), one had an MIC of 16 mg/L, one an MIC of 4 mg/L, two had MICs of 2 mg/L, one had an MIC of 0.5 mg/L, and twelve exhibited MICs ≤ 0.125 mg/L, indicating varying levels of reduced susceptibility (Table S1).

### 3.3. ERG1 Mutations

According to the specific mutations associated with terbinafine resistance, isolates V245-81, n.89, 23-0081, 23-0079, 18 INDIA, and isolate 12 showed 100% homology with the GenBank ID OM313310.1. These isolates share the F397L mutation, a known marker of terbinafine resistance [26,44]. *T. indotineae* isolates 11, 15, and 23-0078 showed a 100% homology with GenBank ID OL415218.1, which represent isolates that lack known *ERG1* mutations [23]. Isolates UCSC 2227 and UCSC TMR reported 100% homology with GenBank ID OL415221.1, suggesting that these isolates harbor the L393S mutation [23]. Isolate 26 matched with OP883944.1, indicating that this is the only strain that carries the F415C mutation [26]. Isolates 13, 14, 15 INDIA, 23-0022, 23-0025, 23-0080, 27, 622/P/23, 633/P/23, and 658/P/23 matched with GenBank ID OL415222.1, the *T. indotineae* reference strain that carries the A448T mutation, a rare terbinafine resistance marker [23,24,25]. Isolate 23-0065 did not exhibit 100% homology with any reference strain in GenBank due to a single nucleotide variation, A900G. However, this substitution did not result in an amino acid change in the Erg1 protein sequence. The relationship between terbinafine MIC values and *ERG1* mutations is summarized in Table 1.

### 3.4. MALDI-TOF Spectra and Machine Learning Analysis

Traditional identification using MALDI-TOF mass spectrometry matching against the Autof MS2600 library indicated that the system could not reliably distinguish *T. indotineae* from *T. mentagrophytes*, with multiple identifications showing overlapping scores although reporting very high values (above 9.0). Several spectra were assigned to *T. interdigitale* or *T. tonsurans*, further highlighting classification ambiguities at the species level.

**Figure 1:**
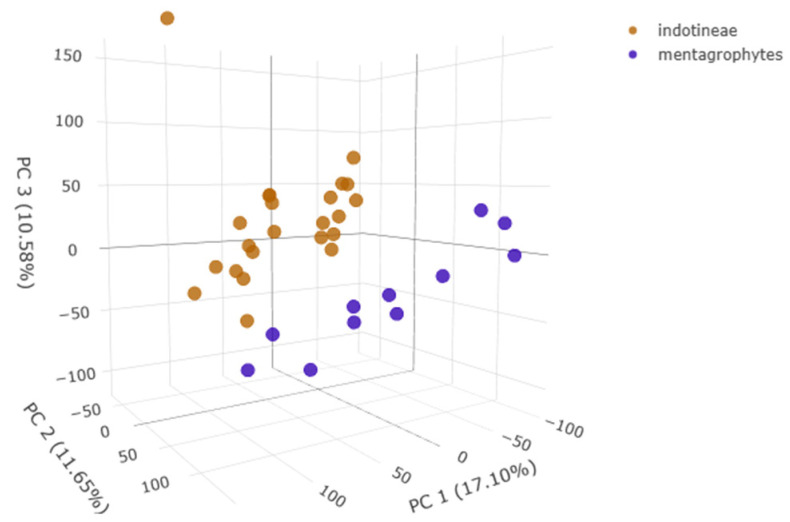
PLS-DA plot of *Trichophyton* spp. spectra.

The integration of MALDI-TOF mass profiles and machine learning analysis allows for improving species discrimination. In particular, the PLS-DA plot (Figure 1) created on the Clover MSDAS platform allowed us to visualize the distribution of *Trichophyton* isolates based on their spectral profiles. The analysis revealed distinct clustering, with *T. indotineae* (orange) and *T. mentagrophytes* (blue) forming separate groups along PC1, PC2, and PC3, indicating clear spectral differentiation.

**Figure 2:**
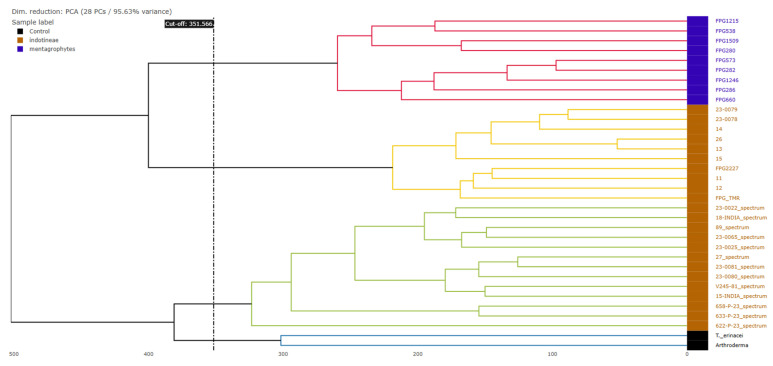
Hierarchical clustering dendrogram based on PCA.

The hierarchical clustering dendrogram based on the MALDI-TOF mass spectra profiles (Figure 2) demonstrated the separation of *T. indotineae* (orange) and *T. mentagrophytes* (blue) species into well-defined clusters (highlighted in different colors basing on the cut-off value). Notably, *Arthroderma* and *T. erinacei* (black) species clustered separately from the *Trichophyton* isolates, confirming their distinct spectral profiles. Within *T. indotineae*, subclusters were visible, indicating potential intra-species spectral variability, although no correlation can be found with the *ERG1* gene mutations observed in the study.

**Figure 3:**
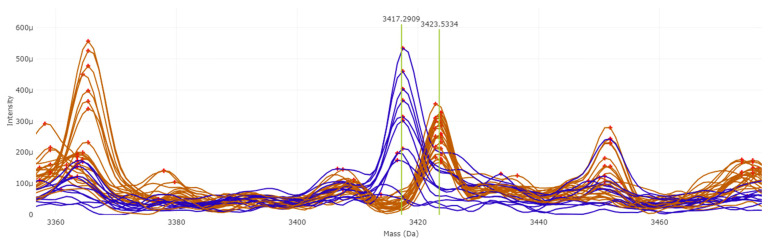
Potential biomarkers for *T. indotineae* (in brown) and *T. mentagrophytes* (in blue).

Trained supervised algorithms yielded a balanced accuracy ranging from 88.74% to 100% (Table S3). Among them, the PLS-DA and SVM algorithms achieved the best results, with 100% balanced accuracy using 10-fold cross validation as the internal validation method. Peaks at 3417.29 and 3423.53 *m*/*z* were found to be putative positive biomarkers for *T. mentagrophytes* and *T. indotineae*, respectively (Figure 3). The 3417.29 *m*/*z* peak shows an AUC of 0.9091 in the ROC curve, while the 3423.53 *m*/*z* peak achieves an AUC of 1. The q-values from the T-test are 2.375 × 10^−2^ and 1.947 × 10^−13^, respectively.

## 4. Discussion

The emergence of *T. indotineae* as a major etiological agent of terbinafine-resistant dermatophytosis has introduced significant challenges in both clinical management and laboratory diagnostics. The molecular characterization of these isolates confirmed the presence of key *ERG1* mutations, particularly F397L, L393S, and A448Y, which have been previously associated with high MIC values and terbinafine treatment failures [24,26] and of which recent whole-genome sequencing studies have provided deeper insights into resistance mechanisms and genetic variability among terbinafine-resistant and -susceptible isolates across different hosts [45]. The increasing global dissemination of *T. indotineae*, first identified in India, has led to outbreaks in Europe, the Middle East, and North America [12]. The widespread and often inappropriate use of topical corticosteroid–antifungal combinations has contributed to selective pressure favoring resistance, leading to persistent, recalcitrant infections that fail conventional treatments [19]. While terbinafine remains a first-line antifungal, its decreasing efficacy necessitates the exploration of alternative therapies, such as azole-based treatments (itraconazole and voriconazole) or combination therapies [1,29]. Future research should focus on evaluating novel antifungal compounds and assessing alternative treatment regimens for *T. indotineae*-associated infections. Clinical presentation of *T. indotineae*-induced dermatophytosis is often severe and extensive, frequently affecting large body areas with hyperkeratotic and inflammatory lesions that pose a therapeutic challenge [13]. Molecular identification remains critical for distinguishing *T. indotineae* from closely related species within the *T. mentagrophytes* complex. To date, ITS sequencing remains the most reliable method for species identification, while *ERG1* sequencing is essential for detecting resistance-associated mutations [28]. However, these techniques require specialized molecular equipment and expertise, making them inaccessible to many routine diagnostic laboratories.

MALDI-TOF mass spectrometry, despite being a widely used rapid fungal identification tool, demonstrated limited accuracy in differentiating *T. indotineae* from *T. mentagrophytes* due to spectral overlap. This is consistent with previous reports, which indicate that current reference libraries lack sufficient spectral resolution to reliably distinguish between these species [11,23]. In response to these limitations, we explored the application of PCA and hierarchical clustering on MALDI-TOF spectral data, which significantly improved species classification. PCA revealed distinct clustering of *T. indotineae* isolates, particularly those harboring terbinafine resistance mutations, highlighting the potential for multivariate analysis to enhance MALDI-TOF-based identification [25]. These findings underscore the urgent need for integrating machine learning and multivariate statistical models into fungal diagnostics. The use of data-driven classification tools could optimize MALDI-TOF-based identification and provide a more accessible and cost-effective alternative to molecular sequencing in resource-limited settings [20]. In fact, despite the initial cost for a mass spectrometer instrument, once available, performing mass profile acquisition implies reduced costs (a few euro cents). Moreover, we were able to discover two mass peaks at 3417.29 and 3423.53 *m*/*z* found to be putative positive biomarkers for *T. mentagrophytes* and *T. indotineae*, respectively. This important achievement might be crucial for improving *T. indotineae* diagnostic strategies and allow for earlier detection of this resistant emerging fungus from this point forward. However, one limitation of this study is the relatively small sample size (n = 56), which restricted the possibility of performing external validation and limited our ability to assess whether the identified biomarkers are broadly representative of the species. The lack of an independent validation set also limits the generalizability of our results, particularly in the context of clinical heterogeneity and geographic variation in *T. indotineae* isolates. Future studies involving larger, multicenter collections of well-characterized isolates from diverse geographical origins will be essential to confirm the validity and robustness of these spectral signatures. Such efforts will also allow for prospective evaluation of the proposed machine learning classifiers in real-world diagnostic workflows, which is a critical step toward their clinical implementation and integration into routine fungal identification practices. Additionally, expanding reference spectral libraries to include high-quality *T. indotineae* isolate profiles will be essential for improving diagnostic accuracy. Given the global rise in terbinafine-resistant *T. indotineae*, our study highlights the need for revised diagnostic strategies.

## 5. Conclusions

*T. indotineae* isolates exhibited variable susceptibility, ranging from wild-type strains with low MICs to highly resistant strains (MIC > 32 µg/mL) harboring well-known mutations such as F397L, A448T, and L393S. While *ERG1* mutations represent the primary mechanism of terbinafine resistance in *T. indotineae*, the heterogeneous MIC values observed in the study among isolates carrying the same substitutions suggest that additional genetic or regulatory factors may contribute to resistance phenotypes. Future studies integrating whole-genome sequencing, transcriptomic profiling, and functional assays will be crucial to uncover alternative resistance pathways, such as efflux pump overexpression or compensatory mutations in sterol biosynthesis. Molecular sequencing remains the gold standard for species identification and resistance detection, while machine learning-assisted spectral analysis presents a promising approach for improving MALDI-TOF-based diagnostics. Strengthening surveillance programs and developing alternative antifungal strategies will be crucial in addressing the rising threat of *T. indotineae* and ensuring effective management of terbinafine-resistant dermatophytosis.

## Figures and Tables

**Table 1:** Distribution of *T. indotineae* terbinafine MIC values (mg/L) by *ERG1* mutation.

*ERG1* Mutation	Sample n.	Mean	MIC Median	Modal
A448T	10	6.49	0.125	0.125
F397L	6	17.33	18.0	32.0
F415C	1	0.5	0.5	0.5
L393S	2	24.0	24.0	16.0
WT	4	0.125	0.125	0.125

## Data Availability

The original contributions presented in the study are included in the article; further inquiries can be directed to the corresponding author.

## Supplementary Materials

The following supporting information can be downloaded at: https://www.mdpi.com/article/10.3390/pathogens14100986/s1, Figure S1. *Trichophyton mentagrophytes*/*interdigitale* phylogenetic tree based on ITS rDNA calculated by neighbor-joining (NJ) algorithm and bootstrap test (1000 replicates), including reference sequences (https://github.com/Ivan-Pchelin/genotyping-by-sequencing/blob/master/referenceset.fasta, accessed on 12 September 2025). Table S1. *Trichophyton* isolates (56) analyzed in this study, including species identification, geographic origin, and minimum inhibitory concentrations (MICs) of terbinafine and *ERG1* mutations. Table S2. Specific hyperparameters used for each of the machine learning algorithms applied in the classification task. Table S3. Performance comparison of the five classification algorithms in identifying *T. mentagrophytes* and *T. indotineae*, showing per-class accuracy and balanced accuracy.
