# Supplementary material for: Integrating Machine Learning and Molecular Methods for Trichophyton indotineae Identification and Resistance Profiling Using MALDI-TOF Spectra
Source: Pathogens. 2025 Sep 30;14(10):986. doi: 10.3390/pathogens14100986 (PMC12567187; doi:10.3390/pathogens14100986)
Supplement: Supplementary file 1 [file pathogens-14-00986-s001.zip › Table S1rev.pdf]

Table S1. *Trichophyton* isolates (56) analyzed in this study, including species identification, geographic origin, minimum inhibitory concentrations (MICs) of terbinafine and ERG1 mutations.

Sample ID	Species	Origin	MIC Terbinafine	ERG1 Mutations
268	*T. mentagrophytes*	FPG	≤0,125	–
280	*T. mentagrophytes*	FPG	≤0,125	–
282	*T. mentagrophytes*	FPG	≤0,125	–
286	*T. mentagrophytes*	FPG	≤0,125	–
359	*T. mentagrophytes*	FPG	≤0,125	–
410	*T. mentagrophytes*	FPG	≤0,125	–
532	*T. mentagrophytes*	FPG	≤0,125	–
538	*T. mentagrophytes*	FPG	≤0,125	–
573	*T. mentagrophytes*	FPG	≤0,125	–
602	*T. mentagrophytes*	FPG	≤0,125	–
660	*T. mentagrophytes*	FPG	≤0,125	–
730	*T. mentagrophytes*	FPG	≤0,125	–
743	*T. mentagrophytes*	FPG	≤0,125	–
751	*T. mentagrophytes*	FPG	≤0,125	–
892	*T. mentagrophytes*	FPG	≤0,125	–
1053	*T. mentagrophytes*	FPG	≤0,125	–
1054	*T. mentagrophytes*	FPG	≤0,125	–
1070	*T. mentagrophytes*	FPG	≤0,125	–
1106	*T. mentagrophytes*	FPG	≤0,125	–
1215	*T. mentagrophytes*	FPG	≤0,125	–
1229	*T. mentagrophytes*	FPG	≤0,125	–
1246	*T. mentagrophytes*	FPG	≤0,125	–
1267	*T. mentagrophytes*	FPG	≤0,125	–
1282	*T. mentagrophytes*	FPG	≤0,125	–
1330	*T. mentagrophytes*	FPG	≤0,125	–
1344	*T. mentagrophytes*	FPG	≤0,125	–
1509	*T. mentagrophytes*	FPG	≤0,125	–
1540	*T. mentagrophytes*	FPG	≤0,125	–
1653	*T. mentagrophytes*	FPG	≤0,125	–
1884	*T. mentagrophytes*	FPG	≤0,125	–
1885	*T. mentagrophytes*	FPG	≤0,125	–
2081	*T. mentagrophytes*	FPG	≤0,125	–
2156	*T. mentagrophytes*	FPG	≤0,125	–
2227	*T. indotineae*	FPG	>32	L393S
TMR	*T. indotineae*	FPG	16	L393S
TMV	*T. mentagrophytes*	FPG	≤0,125	–
15 INDIA	*T. indotineae*	India	≤0,125	A448T
18 INDIA	*T. indotineae*	India	>32	F397L
622/P/23	*T. indotineae*	India	>32	A448T
633/P/23	*T. indotineae*	India	≤0,125	A448T
658/P/23	*T. indotineae*	India	>32	A448T
V245-81	*T. indotineae*	India	>32	F397L
11	*T. indotineae*	Italy	≤0,125	WT
12	*T. indotineae*	Italy	>32	F397L
13	*T. indotineae*	Italy	≤0,125	A448T
14	*T. indotineae*	Italy	≤0,125	A448T

15	*T. indotineae*	Italy	≤0,125	WT
26	*T. indotineae*	Italy	0.5	F415C
27	*T. indotineae*	Italy	≤0,125	A448T
89	*T. indotineae*	Italy	4	F397L
23-0022	*T. indotineae*	Italy	≤0,125	A448T
23-0025	*T. indotineae*	Italy	≤0,125	A448T
23-0065	*T. indotineae*	Italy	≤0,125	WT
23-0078	*T. indotineae*	Italy	≤0,125	WT
23-0079	*T. indotineae*	Italy	2	F397L
23-0080	*T. indotineae*	Italy	0.06	A448T
23-0081	*T. indotineae*	Italy	2	F397L

FPG: Fondazione Policlinico Universitario "A.Gemelli", Rome; India: University of Delhi, Delhi; Italy: Vicenza Hospital, WT: wild-type
