# Supplementary material for: Integrating Machine Learning and Molecular Methods for Trichophyton indotineae Identification and Resistance Profiling Using MALDI-TOF Spectra
Source: Pathogens. 2025 Sep 30;14(10):986. doi: 10.3390/pathogens14100986 (PMC12567187; doi:10.3390/pathogens14100986)
Supplement: Supplementary file 1 [file pathogens-14-00986-s001.zip › Table S2.pdf]

Table S2. Specific hyperparameters used for each of the machine learning algorithms applied in the classification task.

Algorithm	Hyperparameters
**PLS-DA**	Components: 3
	Scale data: Yes
	Positive Category: *T. indotineae*
	Cross Validation: 10-fold
**Random Forest**	Estimators: 50
	Max Depth: 10
	Max Features: 157
	Min Split Size: 2
	Min Samples per Leaf: 1
**SVM**	C: 1
**LightGBM**	Number of Estimators: 100
	Learning Rate: 0.01
	Number of Leaves: 8
	Minimum Child Samples: 3
**KNN**	Number of Neighbors: 3

RF: Random Forest; LightGBM: Light Gradient-Boosting Machine; SVM: Support Vector Machine; PLS-DA: Partial Least Squares Discriminant Analysis; KNN: K-nearest neighbors
