# Supplementary material for: Integrating Machine Learning and Molecular Methods for Trichophyton indotineae Identification and Resistance Profiling Using MALDI-TOF Spectra
Source: Pathogens. 2025 Sep 30;14(10):986. doi: 10.3390/pathogens14100986 (PMC12567187; doi:10.3390/pathogens14100986)
Supplement: Supplementary file 1 [file pathogens-14-00986-s001.zip › Table S3.pdf]

Table S3. Performance comparison of the five classification algorithms in identifying *T. mentagrophytes* and *T. indotineae*, showing per-class accuracy and balanced accuracy.

**Algorithm**	***n*. correct ID /total *n* (% correct ID)**		**Balanced accuracy (%)**
	T. mentagrophytes	*T. indotineae*	
**RF**	9/11 (81.82%)	23/23 (100%)	90.91%
**LightGBM**	9/11 (81.82%)	22/23 (95.65%)	88.74%
**SVM**	11/11 (100%)	23/23 (100%)	100%
**PLS-DA**	10/11 (90.91%)	23/23 (100%)	95.45%
**KNN**	9/11 (81.82%)	23/23 (100%)	90.91%

ID: Identification; RF: Random Forest; LightGBM: Light Gradient-Boosting Machine; SVM: Support Vector Machine; PLS-DA: Partial Least Squares Discriminant Analysis; KNN: K-nearest neighbors
