# Supplementary figures and images for: Integrating Machine Learning and Molecular Methods for Trichophyton indotineae Identification and Resistance Profiling Using MALDI-TOF Spectra
Source: Pathogens. 2025 Sep 30;14(10):986. doi: 10.3390/pathogens14100986 (PMC12567187; doi:10.3390/pathogens14100986)

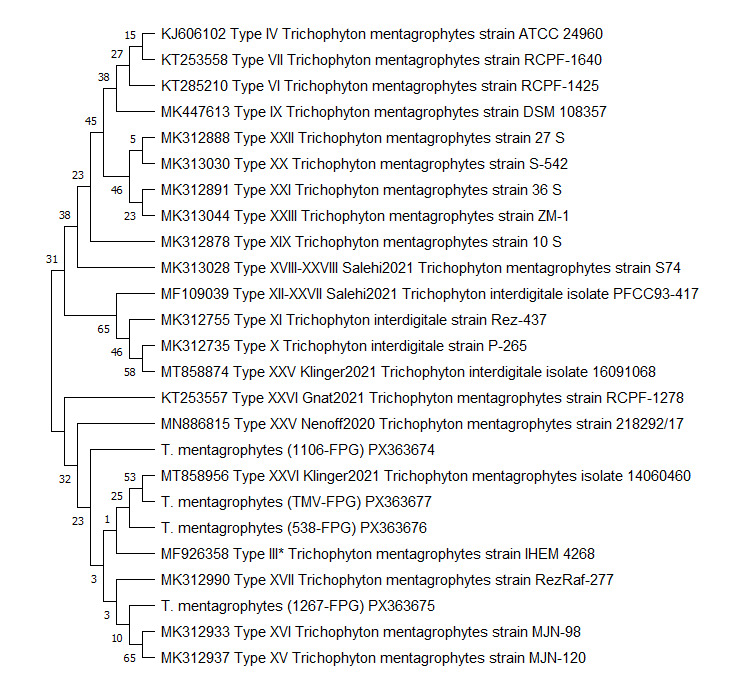

Supplement: Supplementary file 1 [file pathogens-14-00986-s001.zip › FigureS1.tif]
